# Validation of an EMR algorithm to measure the prevalence of ADHD in the Canadian Primary Care Sentinel Surveillance Network (CPCSSN)

**DOI:** 10.1186/s12911-020-01182-2

**Published:** 2020-07-20

**Authors:** Rachael Morkem, Kenneth Handelman, John A. Queenan, Richard Birtwhistle, David Barber

**Affiliations:** 1 Research Associate, Centre for Studies in Primary Care, Queen’s University, Kingston, Ontario Canada; 2 Psychiatrist, Centre for Integrative Mental Health, Oakville, Ontario Canada; 3 Senior Epidemiologist, Centre for Studies in Primary Care, Queen’s University, Kingston, Ontario Canada; 4 Professor of Family Medicine and Public Health Sciences, Centre for Studies in Primary Care, Queen’s University, Kingston, Ontario Canada; 5 Network Director and Assistant Professor, Centre for Studies in Primary Care, Queen’s University, Kingston, Ontario Canada

**Keywords:** Validation, Algorithm, Attention deficit-hyperactivity disorder, Prevalence

## Abstract

**Background:**

Building and validating electronic algorithms to identify patients with specific disease profiles using health data is becoming increasingly important to disease surveillance and population health management***.*** The aim of this study was to develop and validate an algorithm to find patients with ADHD diagnoses within primary care electronic medical records (EMR); and then use the algorithm to describe the epidemiology of ADHD from 2008 to 2015 in a Canadian Primary care sample.

**Methods:**

This was a cross sectional time series that used data from the Canadian Primary Care Sentinel Surveillance Network (CPCSSN), a repository of primary care EMR data. A sample of electronic patient charts from one local clinic were manually reviewed to determine the positive predictive value (PPV) and negative predictive value (NPV) of an ADHD case-finding algorithm. In each study year a practice population was determined, and the algorithm was used to measure an observed prevalence of ADHD. The observed prevalence was adjusted for misclassification, as measured by the validity indices, to obtain an estimate of the true prevalence. Estimates were calculated by age group (4–17 year olds, 18 to 34 year olds, and 35 to 64 year olds) and gender, and compared over time.

**Results:**

The EMR algorithm had a PPV of 98.0% (95% CI [92.5, 99.5]) and an NPV of 95.0% (95% CI [92.9, 98.6]). After adjusting for misclassification, it was determined that the prevalence of patients with a clinical diagnosis of ADHD has risen in all age groups between 2008 and 2015, most notably in children and young adults (6.92, 95% CI [5.62, 8.39] to 8.57, 95% CI [7.32, 10.00]; 5.73, 95% CI [4.40, 7.23] to 7.33, 95% CI [6.04, 8.78], respectively). The well-established gender gap persisted in all age groups across time but was considerably smaller in older adults compared to children and young adults.

**Conclusion:**

Overall, the ADHD case-finding algorithm was found to be a valid tool to assess the epidemiology of ADHD in Canadian primary care practice. The increased prevalence of ADHD between 2008 and 2015 may reflect an improvement in the recognition and treatment of this disorder within primary care.

## Background

Attention Deficit Hyperactivity Disorder (ADHD) is the most commonly diagnosed mental disorder in children and has a significant impact on an individual’s development, education and socialization throughout their life course [1, 2]. Longitudinal studies are now finding that the symptoms, once thought to remit beyond puberty, persist well into adulthood in 58 to 70% of individuals [3, 4]. When untreated this disorder is associated with under achievement in school, lower occupational achievement, harm to relationships with family, teachers and friends, increased criminality and accidents, and development of comorbid psychiatric symptoms [1–3, 5].

ADHD is one of the three most common disorders seen in primary care. As such, primary care providers are central to treating this condition [6]. This can be challenging for general practitioners as they often have limited formal training in mental health concerns in children and adolescents, which is commonly the age at which this condition is first recognized 6). The first-line treatment for ADHD are central nervous system stimulants, specifically methylphenidate, which has been used for more than 50 years, and amphetamines [5]. Other non-stimulant medications include atomoxetine and extended release guanfacine, a selective alpha-agonist [2]. In addition, there is evidence that some antipsychotics are being prescribed, off-label, to treat ADHD [7–12].

Recently, several studies have been published that evaluated the prevalence of ADHD using Canadian healthcare data. An Ontario study by Hauck et al. manually reviewed electronic medical records and applied predetermined criteria to identify ADHD cases [12]. They found a prevalence of 5.4% (7.9% in males and 2.7% in females) in youth aged 1 to 24 years [12]. Another study used administrative data to evaluate temporal trends in ADHD diagnoses by province [13]. In this study the ADHD case definition relied on physician diagnosis and hospital admissions and found the 2011–2012 prevalence in Ontario was 16.0 per 1000 in males and 5.8 per 1000 in females [13]. A study published in 2018 used survey data from a large national sample of Canadian adults and found a prevalence of 2.9% in adults, 20 to 64 years of age [14].

Measuring and evaluating the primary care of children and adults with ADHD is important to promote their health and improve their long-term outlooks. As such, it is important to develop methods of detecting patients with an established ADHD diagnosis within the electronic medical record (EMR). The objectives of this study were to develop and validate an EMR case definition of ADHD diagnosis within a novel national dataset and report on the epidemiology of this disorder in children and adults within primary care.

Ethics approval was granted by Health Sciences and Affiliated Teaching Hospitals Research Ethics Board at Queen’s University, Kingston.

## Methods

### Description of data source

This study used Canadian primary health care data. Canada has a publically funded health care system where primary care providers are self-employed in private practices and act as gate-keepers to other specialists [15]. While healthcare is covered by public insurance, prescription drug costs must be paid out-of-pocket by patients. However, various provincial drug plans cover specific populations (ex. seniors) and many have private insurance through employers [15]. The data for this project came from the Canadian Primary Care Sentinel Surveillance Network (CPCSSN). CPCSSN is a pan-Canadian organization made up of 11 practice based research networks (PBRN) in eight provinces that extracts primary care clinical data from electronic medical records (EMR). Primary care providers agree to have their patients’ data extracted, stripped of identifiers and aggregated into a large database to be used for research and surveillance. The database includes demographic and clinical information including diagnoses, billing codes, prescriptions and labs. Patients with records within the CPCSSN database are somewhat representative of the Canadian general population but are older and more likely to be women [16]. This is because older individuals are more likely to have healthcare needs than younger individuals and women are more likely to seek help for healthcare problems, thus leading to an over representation of these patients in the database compared to the general population [16]. At the time of the study the CPCSSN database included 1.5 million patients across Canada.

This study only included data from six of the 11 PBRNs that comprise CPCSSN due to limitations on data usage for industry funded projects. The data included in this study came from networks located in the provinces of Alberta, Manitoba, Ontario, Quebec and Newfoundland.

### Study population

In order to estimate the prevalence of patients who receive a clinical diagnosis of ADHD it is essential to determine the population at risk. This means identifying a practice population, individuals that are active patients and receive the majority of their primary care from the same clinic. This study used a two-year contact group to define the yearly practice populations. In each year of study (2008 to 2015), the practice population consisted of any patient with an encounter in the year of study or the preceding year. This method of using two years ofdata to determine a yearly practice population has been shown to most effectively capture the population at risk [17].

Only patients with a valid entry for year of birth and gender were included.

### Attention deficit hyperactivity disorder (ADHD) case definition

A patient was classified as having ADHD if they met the case definition for this disorder. A case definition was created and validated using a manual chart abstraction. The case definition, adapted from Kirby et al., used the medication, billing, diagnosis and encounter tables within the CPCSSN database to classify patients with ADHD [18]. A patient was classified as having ADHD if they were 4 years of age or older and either (a) the medical record included ICD-9 code 314 in one or more visits, and one or more prescriptions of ADHD-related medications; or (b) the medical record included ICD-9 code 314 in two or more visits. Patients were excluded if they had ICD9 codes corresponding to exclusionary medcal conditions (Additional file 1). The algorithm was validated by conducting a manual electronic chart review of a sample of 492 patients by a blinded abstractor. This sample was comprised of charts from a single clinic of all the ADHD cases detected by the algorithm (*n* = 246) and a random sample of patients (n = 246) not detected as having ADHD by the algorithm. The sample of subjects without ADHD were not age or sex matched to the subjects with ADHD as this algorithm was designed to capture ADHD diagnoses in all age groups (≥4 years of age) and both genders. While sensitivity and specificity could not be directly measured, as the validation sample was not a random selection of patients, we could get direct measures of the positive predictive value (PPV) and the negative predictive value (NPV) for our EMR algorithm. The sensitivity and specificity of the algorithm were derived using analytical expressions based on the Rogan-Gladen estimator [19, 20]. The positive predictive value (PPV) of the algorithm was calculated by determining the number of patients classified as having ADHD by both the algorithm and the chart abstractor, out of the all the patients classified as having ADHD by the algorithm. The negative predictive value (NPV) of the algorithm was calculated by determining the number of patients classified as not having ADHD by both the algorithm and the chart abstractor out of all the patients classified as not having ADHD by the algorithm. Any patient in the sample (*n* = 492) that had a discordant ADHD classification between the algorithm and the chart abstractor was reviewed by the study epidemiologist to determine the source of misclassification.

### Statistical analysis

The case definition was applied to each yearly practice population to produce a count of those with a clinical diagnosis of ADHD. This yearly count was used to determine the observed prevalence, defined as the proportion of the practice population that met the ADHD criteria. To obtain an estimate of the true prevalence in the database population the observed prevalence was adjusted for misclassification using the validity indices (NPV and PPV) determined by the validation of the chart algorithm. The analytical expressions used to calculate the true prevalence were recently published in a study by Bollaerts et al., and are available as a user-friend web application [19].

Patients were classified into three age groups: 4–17 year olds, 18–34 year olds, and 35–64 year olds. These categories were chosen because the diagnosis and treatment of ADHD differs for school-age children and adults. In addition, young adulthood marks a distinct transition period in a person’s life course [21, 22]. Seniors were excluded because of the low ADHD prevalence in this age group.

The prevalence and corresponding confidence limits were computed using an exact binomial test. The analysis was performed in SAS statistical software (version 9.6®). The online application developed by Bollaerts et al. was used as the analytical tool to calculated adjusted sensitivity and specificity and estimate true prevalence values [19].

## Results

Between 2008 and 2015 the practice population in each study year, of those at least 4 years of age, increased from 345,173 to 624,419 (Table 1). This increase is due to the growing uptake of EMRs and the surge in number of practices contributing patient data to CPCSSN.

**Table 1:** Study population at risk (yearly)

	2008	2009	2010	2011	2012	2013	2014	2015
**n**	345,173	413,715	460,970	528,698	565,517	587,707	609,756	624,419
**Age, %**								
4–17 years	18.5	18.8	18.4	18.3	18.5	18.6	18.6	18.7
18–34 years	25.6	25.9	26.1	26.4	26.6	26.8	26.8	26.8
35–64 years	55.9	55.3	55.5	55.3	54.8	54.7	54.6	54.5
**Gender, %**								
male	42.0	42.4	42.9	43.0	43.1	43.1	43.3	43.6

The EMR algorithm, described in Table 2, identified 246 patients with ADHD within a single clinic that has records of 19,683 patients. Using the algorithm, a random sample of 246 patients who did not meet the criteria for ADHD were identified. The blinded chart abstractor analyzed 492 charts: all 246 patients identified by the algorithm as having ADHD, and 246 patients identified by the algorithm as not having ADHD. After a thorough examination of all documentation within the medical chart the abstractor found that 236 of the 246 patients that the algorithm identified as ADHD cases had an ADHD diagnosis (true positives); and 237 of the 246 patients that the algorithm identified as not having ADHD had no ADHD diagnosis (true negatives). An evaluation of the patients with discordant diagnoses (algorithm and chart abstractor in disagreement) revealed that most of the ADHD cases missed by the algorithm (*n* = 9) were due to management of the disorder by a specialist, and there was subsequently very little documentation (billings, encounter diagnoses, medications) within the primary care EMR. The chart abstractor found record of the ADHD diagnosis within free text notes in the medical history or referral letters, which is data that isn’t captured within the CPCSSN database. The patients misclassified by the algorithm as having ADHD (*n* = 10) were almost all subjects where a provider had evaluated a patient for ADHD but a diagnosis or treatment plan did not follow. From these results, it was determined that the ADHD algorithm has a PPV of 95.9% (95% CI [92.6, 98.0]) and an NPV of 96.3% (95% CI [93.2, 98.3]). Using the analytic expressions developed by Bollaerts et al., the derived sensitivity is 19.9% (95% CI [15.9, 25.6]) and the derived specificity is 100.0% (95% CI [99.9, 100.0]) [19].

**Table 2:** Case definition for ADHD

Inclusion Criteria	
individual is 4 years old or older; **and**	
individual’s medical record includes ICD9 code in one or more in-person visits, on separate calendar days; **and**	
individual’s medical record includes one or more prescriptions of ADHD-related medications;	
**Or**	
individual is 4 years old or older; **and**	
individual’s medical record includes a relevant ICD9 code (314) on two or more in-person visits, on separate calendar days	
Exclusion Criteria	
Individual’s medical record includes one or more ICD9 codes corresponding to an exclusionary medical condition (see Additional file 1).	

This case definition was used to measure the observed prevalence of patients with a clinical diagnosis of ADHD in each yearly practice population. Table 3 provides estimates of the true prevalence (observed prevalence adjusted for misclassification), by age group. In each study year the prevalence was highest in children and youth 4 to 17 years old. However, the prevalence in young adults (18 to 34 years old) also rose dramatically, and by 2015 it approaches the estimate observed in children. There was a slight increase in the prevalence of a clinical diagnosis of ADHD in adults 35 to 64 years old.

**Table 3:** Prevalence of ADHD, by age

Year	Age Group % of patients (95% CI)		
	4–17	18–34	35–64
2008	6.92 (5.62, 8.39)	5.73 (4.40, 7.23)	5.20 (3.87, 6.73)
2009	6.99 (5.69, 8.49)	5.88 (4.57, 7.36)	5.22 (3.93, 6.74)
2010	7.35 (6.08, 8.81)	6.07 (4.76, 7.56)	5.27 (3.95, 6.75)
2011	7.63 (6.36, 9.08)	6.24 (5.00, 7.77)	5.32 (4.02, 6.81)
2012	7.87 (6.61, 9.37)	6.48 (5.18, 7.98)	5.36 (4.03, 6.89)
2013	8.14 (6.89, 9.60)	6.74 (5.45, 8.23)	5.43 (4.12, 6.93)
2014	8.40 (7.13, 9.86)	7.05 (5.74, 8.52)	5.48 (4.17, 7.00)
2015	8.57 (7.32, 10.00)	7.33 (6.04, 8.78)	5.54 (4.22, 7.02)

*CI* Confidence interval

**Fig. 1:**
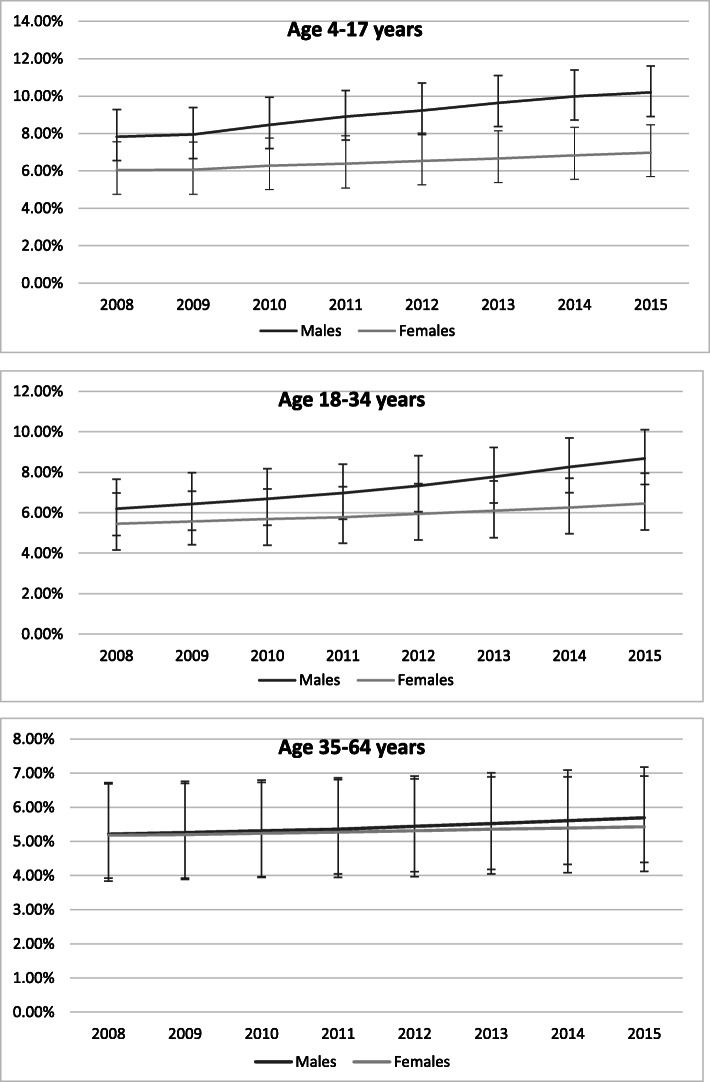
Prevalence of ADHD by age and sex

As expected, there was a large gender gap in the prevalence of diagnosed ADHD in the younger age groups (Fig. 1). In children and youth the ratio of male to female ADHD diagnoses increased slightly from 1.3:1 in 2008 to 1.4:1 by 2015. In contrast, in young adults the gap widened from 1.1:1 to 1.3:1. In 35 to 64 year olds the gender gap remained very low across all study years.

## Discussion

This study developed and validated a method for identifying patients with established ADHD diagnoses using EMR primary care data in Canada. The algorithm has a PPV of 98.0% and an NPV of 95.0%. These measures of the validity of the algorithm allowed us to adjust the observed prevalence for misclassification and obtain estimates of the true prevalence of ADHD within the database population. Consequently, we can have confidence that applying the case definition to the EMR data captured within the CPCSSN database, and adjusting for misclassification, is an effective method to evaluate the prevalence of ADHD within primary care.

This algorithm can be used to estimate the burden that this disease has on the population so that resources and interventions can be allocated where there is need. Furthermore, with expected developments in natural language processing, it is hoped that the free text within EMRs could be used to augment the algorithm. This means the algorithms would be more clinically relevant and could be used to screen a provider’s practice and find patients at risk for ADHD, or those that may have it and are not yet diagnosed.

Between 2008 and 2015, the prevalence of patients with a clinical diagnosis of ADHD rose dramatically in children (4–17 years) and young adults (18–34 years). As expected, the prevalence of ADHD diagnoses is higher amongst children and young adults compared to older adults (35–64 years). Previous studies, including a systematic review and meta-analysis, have shown that the actual prevalence of ADHD is not increasing over time, which provides evidence that there is an increase in the identification and diagnosis of ADHD in primary care between 2008 and 2015, rather than an increase of this disorder in the population [7, 12, 21, 23]. It is thought that this increased identification of ADHD is due to changes in diagnostic criteria, education policies and increasing awareness and access to medical services [23, 24]. The rise in ADHD diagnoses in the younger age groups may reflect the fact that ADHD has historically been recognized as a disorder of childhood and clinicians may not recognize it if the condition is on setting in adulthood [25, 26].

A recent study by Vasiliadis et al. used routine epidemiologic data from four provinces (Ontario, Manitoba, Quebec and Nova Scotia) to evaluate temporal trends and found a significant increase in the prevalence of diagnosed ADHD between 1999 and 2012 [13]. While Vasiliadis et al. found significant variation between provinces the overall upward trend is consistent with our findings. The study by Hauck et al. that manually reviewed 10,000 electronic charts in 2012, estimated the prevalence of a clinical diagnosis of ADHD in those 1 to 24 years of age at 5.4 [12]. In contrast, the average prevalence of ADHD between 2008 and 2015 in those aged 4 to 17 years in our study is 7.75 (standard deviation, 1.47). This is likely comparable as it is expected our estimate would be slightly higher due to the narrower age range. As well, the lower estimate found by Hauck et al. could be attributed to geographic variation. The study by Hauck et al. used administration data from one large urban center in Ontario and our study used data from urban and rural primary care clinics across Canada [12]. The existence of regional differences is confirmed in the study by Vasiliadis et al. [13].

There was a small increase in the prevalence of ADHD diagnoses in adults 35 to 64 years old. This could be due to an increasing recognition that the symptoms of ADHD often persist into adulthood [14]. However, this study did not determine if these prevalent cases were detection of previously undiagnosed ADHD or the earliest documentation of ADHD in patients with an established diagnosis. There has been some suggestion that ADHD is being over diagnosed in adults due to changes in diagnostic thresholds, poor diagnostic practices, and advertising by pharmaceutical industry [24]. However, these results suggest that, in Canada, the prevalence of adults with a clinical diagnosis of ADHD, especially younger adults, is comparable to the prevalence of adult ADHD recognized in other countries. Kessler et al. found a prevalence of 4.4% in adults 18–44 years, and the World Health Organization (WHO) quotes an adult ADHD rate of 2.8% worldwide [26, 27].

The distinct gender difference in the prevalence of ADHD found in this study is well-established in the literature [28–31]. However, it is noteworthy that the gap is considerably smaller in adults 18 to 34 years old and 35–64 years old. This supports the premise that the gender gap is an artifact of the school system, where boy’s symptomology manifests as disruptive behavior and is more likely to be noticed and diagnosed [27, 28]. A recent study evaluating the gender difference in adult ADHD further confirms this, showing that, despite lower rates of hyperactive symptoms, women and men have similar rates of current ADHD [29]. However, it is only in the last two decades that there has been a shift in the conceptualization of ADHD as a disorder present at any age, and evidence has been building that ADHD is not a predominantly male disorder [28, 29].

There are a number of limitations to this study. Firstly, the case definition for ADHD was validated using a manual chart review from one clinic using one EMR product. While this provides confidence in the robustness of the algorithm it would be ideal to validate this case definition across several EMR products and within multiple clinics. It is possible that the results of the validation may only be generalizable to this clinic using this EMR product. Furthermore, an analysis of the subjects with discordant classifications between the algorithm and chart reviewer (*n* = 19) revealed that most of the missed cases (*n* = 9) were a result of a diagnosis being recorded within free text notes, which is not captured within the CPCSSN database; and most of the falsely identified ADHD cases (*n* = 10) a result of evidence within the EMR (billing codes) of a provider examining a patient with symptoms congruent with ADHD, but not diagnosed with the disorder. While there is no reason to believe this misclassification differs across gender or time the algorithm was designed to capture ADHD in patients of any gender or age (≥4 years) and it is possible the validation measures of the algorithm could differ in specific groups. Lastly, this study used data from a convenience sample of primary care clinics from five provinces. As such, the yearly study populations may not be representative of all Canadians.

## Conclusion

The findings from this study demonstrate the reliability of an ADHD case finding algorithm in a large Canadian primary care database. Applying this algorithm it was determined that the prevalence of patients with a clinical diagnosis of ADHD increased between 2008 and 2015, likely as a result of increased recognition and treatment of this disorder within primary care. There still exists a significant gender gap. Many patients with ADHD rely on their primary care providers for the care and support they need to manage their disorder and its adverse effects. Establishing robust methods to measure and evaluate the care of patients with ADHD is important in order to ensure clinicians are providing the best care and support for their patients.

## Supplementary information

**Additional file 1.**

## Data Availability

The data that support the findings of this study are available from the *Canadian Primary Care Sentinel Surveillance Study (CPCSSN)* but restrictions apply to the availability of this data, which were used under license for the current study, and so are not publicly available. Data are however available from the authors upon reasonable request and with permission of CPCSSN.

## Abbreviations

ADHD: Attention Deficit-Hyperactivty Disorder
PBRN: Practice Based Research Network
EMR: Electronic Medical Record
CPCSSN: Canadian Primary Care Sentinel Surveillance Network
PPV: Positive Predictive Value
NPV: Negative Predictive Value
WHO: World Health Organization
