# Supplementary material for: Validation of an EMR algorithm to measure the prevalence of ADHD in the Canadian Primary Care Sentinel Surveillance Network (CPCSSN)
Source: BMC Med Inform Decis Mak. 2020 Jul 20;20:166. doi: 10.1186/s12911-020-01182-2 (PMC7370518; doi:10.1186/s12911-020-01182-2)
Supplement: Supplementary file 1 — Additional file 1. [file 12911_2020_1182_MOESM1_ESM.docx]

**ADHD PHENOTYPE ALGORITHM**

**Background:** Attention deficit-hyperactivity disorder (ADHD) is a complex neuropsychiatric disorder, with an estimated prevalence of 7% of school-age children and a smaller percentage (~4%) of adults. Three subtypes include predominantly inattentive, predominantly hyperactive-impulsive, and combined hyperactive-inattentive. In ICD-9 and ICD-10, ADHD is listed with hyperkinetic disorder/hyperkinetic syndrome (HKD).

**Cases:** Cases must be 4 years of age or older, and are defined by a diagnostic history of ADHD/HKD as determined by ICD9 codes and/or a history of ADHD medications.

Case inclusion criteria:

1a) Individual is 1460 days-old or older

*plus*

1b) Individual’s medical record includes a relevant ICD9 code (see **Table 1**) in one or more in-person visits, on separate calendar days

*plus*

1c) Individual’s medical record includes one or more prescriptions of ADHD-related medications (see **Table 2**)

*or*

2a) Individual is 1460 days-old or older

*plus*

2b) Individual’s medical record includes a relevant ICD9 code (see **Table 1**) in two or more in-person visits, on separate calendar days.

Case exclusion criteria:

Individual’s medical record includes one or more of the ICD9 codes shown in **Table 3**.

**Controls:** Controls must be 4 years of age, or older. Exclusionary criteria for controls include psychiatric, neurological and related disorders as defined by ICD9 codes or patient chart. Additionally, controls are excluded if his/her medical record includes a medication used to treat psychiatric, neurological and related disorders.

Control inclusion criterion:

1. Individual is 1460 days-old or older.
2. Individual must have two or more visits in the last five years (of last day in record)

Control exclusion criteria:

1) Individual’s medical record includes one or more prescriptions for a medication addressing psychiatric, neurological or related disorders (see **Table 4**)

*and/or*

2) Individual’s medical record includes one or more ICD9 codes addressing psychiatric, neurological or related disorders (see **Table 5**)

*and/or*

3) Individual’s patient chart includes one or mentions of an ADHD or hyperkinesias (see **Table 1**).

**Covariates:** Relevant covariates are: 1) age, 2) gender, 3) ethnicity, race, and 4) birth month (younger children can be more hyperactive in the classroom, which may be mis-diagnosed as ADHD) Ref: Elder Todd. The Importance of Relative standards in ADHD Diagnoses: Evidence Based on Exact Birth Dates. J. Health Economy. Sept 2010;29(5):641-656).

| **Table 1, ADHD Cases: Inclusionary ICD Codes** | |
| --- | --- |
| **ICD-9 Code** | **Diagnosis** |
| **314** | Hyperkinetic syndrome of childhood |
| **314.0** | Attention deficit disorder |
| **314.01** | With hyperactivity |
| **314.1** | Hyperkinesis with developmental delay, Developmental disorder of hyperkinesis |
| **314.2** | Hyperkinetic conduct disorder |
| **314.8** | Other specified manifestations of hyperkinetic syndrome |
| **314.9** | Unspecified hyperkinetic syndrome |

| **Table 2, ADHD Cases: Inclusionary medications by sub-type.** | | |  |
| --- | --- | --- | --- |
| **Stimulants** | **Non-Stimulants** | **Other Drugs** | **Other Drugs** |
| Methylphenidate | Norepinephrine reuptake inhibitor | NDRI | Imipramine |
| Attenta | NRI | Amfebutamone | Melipramine |
| Concerta | Atomoxetine | Aplenzin | Tofranil |
| Daytrana | Attentin | Buproprion | Lithium |
| Desoxyn | Strattera | Elontril | Eskalith |
| Hynidate | Tomoxetin | Prexaton | Lithobid |
| Metadate |  | Voxra | Olanzapine |
| Methylin |  | Wellbutrin | Zyprexa |
| Methyllin | **Alpha-2 Agonists** | Zyban | Paroxetine |
| Rilatine | Clonidine | SNRI's (Tricyclics) | Aropax |
| Ritalin | Guanfacine | Imipramine | Brisdelle |
| Dexmethylphenidate |  | Melipramine | Paxil |
| Attenade |  | Tofranil | Pexeva |
| Celgene |  | Carbamazepine | Sereupin |
| Focalin |  | Equetro | Seroxat |
| Amphetamine |  | Tegretol | Pemoline |
| Adderall |  | Clonazepam | Betanamin |
| Dexedrine |  | Clonex | Ceractiv |
| DextroStat |  | Klonopin | Cylert |
| Prodrug Amphetamines |  | Kriadex | Tradon |
| Lisdexamfetamine (Vyvanse) |  | Linotril | Risperidone |
|  |  | Paxam | Risperdal |
|  |  | Petril | Sertraline |
|  |  | Ravotril | Altruline |
|  |  | Rivatril | Besitran |
|  |  | Rivotril | Daxid |
|  |  | Divalproex | Deprax |
|  |  | Depakote | Eleval |
|  |  | Epilim | Emergen |
|  |  | Fluoxetine | Gladem |
|  |  | Fontex | Implicane |
|  |  | Prozac | Lowfin |
|  |  | Sarafem | Lustral |
|  |  | Hydroxyzine | Sealdin |
|  |  | Alamon | Sedoran |
|  |  | Atarax | Serivo |
|  |  | Aterax | Sertralin |
|  |  | Durrax | Stimuloton |
|  |  | Equipose | Tresleen |
|  |  | Orgatrax | Zoloft |
|  |  | Masmoran | Trazodone |
|  |  | Paxistil | Beneficat |
|  |  | Quiess | Deprax |
|  |  | Tran-Q | Desirel |
|  |  | Tranquizine | Desyrel |
|  |  | Vistaril | Mesyrel |
|  |  |  | Molipaxin |
|  |  |  | Oleptro |
|  |  |  | Thombran |
|  |  |  | Trazorel |
|  |  |  | Trialodine |
|  |  |  | Trittico |
|  |  |  |  |
|  |  |  |  |

| **Table 3, ADHD Cases: Exclusionary ICD9 Codes.** | |
| --- | --- |
| **Psychiatric and Related** | |
| **ICD-9 Codes** | **Diagnosis** |
| 290 | Dementias |
| 294 | Persistent mental disorders due to conditions classified elsewhere- |
| 300.8x | Somatoform disorders |
| 301.51 | Chronic factitious illness with physical symptoms |
| 307.3x | Stereotypic movement disorder |
| 317-317.x | Mild mental retardation |
| 318-318.x | Other specified mental retardation |
| 319-319.x | Unspecified mental retardation |
| **General** | |
| **ICD-9 Codes** | **Diagnosis** |
| 006.5 | Amebic brain abscess |
| 013.2 | Tuberculoma of brain |
| 191-191.x | Malignant neoplasm of brain |
| 192-192.x | Malignant neoplasm of other and unspecified parts of nervous system |
| 237.7x | Neurofibromatosis |
| 290.1 | Presenile dementia |
| 348.1 | Anoxic brain damage |
| 348.2 | Benign intracranial hypertension |
| 348.3 | Encephalopathy, not elsewhere classified |
| 348.4 | Compression of brain |
| 348.5 | Cerebral edema |
| 348.8 | Other conditions of brain |
| 348.9 | Unspecified condition of brain |
| 437.2 | Hypertensive encephalopathy |
| 742-742.x | Other congenital anomalies of nervous system |
| 764-764.x | Slow fetal growth and fetal malnutrition |
| 767.0 | Subdural and cerebral hemorrhage |
| 767.9 | Birth trauma, unspecified |
| 800-804 | Fracture of skull |
| 959.01 | Head injury, unspecified |

| **Table 4, ADHD Controls: Exclusionary medications by sub-type.** | | |  |
| --- | --- | --- | --- |
| **Stimulants** | **Non-Stimulants** | **Other Drugs** | **Other Drugs** |
| Methylphenidate | Norepinephrine reuptake inhibitor | SNRI's (Tricyclics) | Imipramine |
| Attenta | NRI | Imipramine | Melipramine |
| Concerta | Atomoxetine | Melipramine | Tofranil |
| Daytrana | Attentin | Tofranil | Lithium |
| Desoxyn | Strattera | Carbamazepine | Eskalith |
| Hynidate | Tomoxetin | Equetro | Lithobid |
| Metadate |  | Tegretol | Olanzapine |
| Methylin |  | Clonazepam | Zyprexa |
| Methyllin |  | Clonex | Paroxetine |
| Rilatine |  | Klonopin | Aropax |
| Ritalin |  | Kriadex | Brisdelle |
| Dexmethylphenidate |  | Linotril | Paxil |
| Attenade |  | Paxam | Pexeva |
| Celgene |  | Petril | Sereupin |
| Focalin |  | Ravotril | Seroxat |
| Amphetamine |  | Rivatril | Pemoline |
| Adderall |  | Rivotril | Betanamin |
| Dexedrine |  | Divalproex | Ceractiv |
| DextroStat |  | Depakote | Cylert |
| Prodrug Amphetamines |  | Epilim | Tradon |
| Lisdexamfetamine (Vyvanse) |  | Fluoxetine | Risperidone |
|  |  | Fontex | Risperdal |
|  |  | Prozac | Sertraline |
|  |  | Sarafem | Altruline |
|  |  | Hydroxyzine | Besitran |
|  |  | Alamon | Daxid |
|  |  | Atarax | Deprax |
|  |  | Aterax | Eleval |
|  |  | Durrax | Emergen |
|  |  | Equipose | Gladem |
|  |  | Orgatrax | Implicane |
|  |  | Masmoran | Lowfin |
|  |  | Paxistil | Lustral |
|  |  | Quiess | Sealdin |
|  |  | Tran-Q | Sedoran |
|  |  | Tranquizine | Serivo |
|  |  | Vistaril | Sertralin |
|  |  |  | Stimuloton |
|  |  |  | Tresleen |
|  |  |  | Zoloft |
|  |  |  | Trazodone |
|  |  |  | Beneficat |
|  |  |  | Deprax |
|  |  |  | Desirel |
|  |  |  | Desyrel |
|  |  |  | Mesyrel |
|  |  |  | Molipaxin |
|  |  |  | Oleptro |
|  |  |  | Thombran |
|  |  |  | Trazorel |
|  |  |  | Trialodine |
|  |  |  | Trittico |

| **Table 5, ADHD Control Exclusionary ICD9 Codes.** | |
| --- | --- |
| **ICD-9 Codes** | **Diagnosis** |
| 006.5 | Amebic brain abscess |
| 013.2 | Tuberculoma of brain |
| 191-191.x | Malignant neoplasm of brain |
| 192-192.x | Malignant neoplasm of other and unspecified parts of nervous system |
| 237.7x | Neurofibromatosis |
| 290-319.x | Mental disorders |
| 327-327.x | Organic sleep disorders |
| 330-337.x | Hereditary and degenerative diseases of the central nervous system |
| 342-342.x | Hemiplegia and hemiparesis |
| 343-343.x | Infantile cerebral palsy |
| 344-344.x | Other paralytic syndromes |
| 345-345.x | Epilepsy and recurrent seizures |
| 347-347.x | Cataplexy and narcolepsy |
| 348-348.x | Other conditions of brain |
| 349-349.x | Other and unspecified disorders of the nervous system |
| 437.2 | Hypertensive encephalopathy |
| 742-742.x | Other congenital anomalies of nervous system |
| 758.x | Chromosomal anomalies |
| 764-764.x | Slow fetal growth and fetal malnutrition |
| 765-765.x | Disorders relating to short gestation and low birthweight |
| 767.0 | Subdural and cerebral hemorrhage |
| 767.9 | Birth trauma, unspecified |
| 779.4 | Drug reactions and intoxications specific to newborn |
| 779.5 | Drug withdrawal syndrome in newborn |
| 781-781.x | Symptoms involving nervous and musculoskeletal systems |
| 800-804 | Fracture of skull |
| 959.01 | Head injury, unspecified |
| 996.2 | Mechanical complication of nervous system device, implant, and graft |
